# Temporal changes of fine root overyielding and foraging strategies in planted monoculture and mixed forests

**DOI:** 10.1186/s12898-018-0166-z

**Published:** 2018-02-17

**Authors:** Weiwei Shu, Xiaoxiao Shen, Pifeng Lei, Wenhua Xiang, Shuai Ouyang, Wende Yan

**Affiliations:** 1 Faculty of Life Science and Technology, Central South University of Forestry and Technology, Changsha, 410004 Hunan China; 2 National Engineering Laboratory for Applied Technology of Forestry & Ecology in South China, Central South University of Forestry and Technology, Changsha, 410004 Hunan China

**Keywords:** Fine root biomass, Vertical distribution, Specific root length, Overyielding, Stand age

## Abstract

**Background:**

Mixed forests are believed to enhance ecosystem functioning and sustainability due to complementary resource use, environmental benefits and improved soil properties. The facilitation between different species may induce overyielding. Meanwhile, the species-specific fine root foraging strategies and tradeoffs would determine the structure and dynamics of plant communities. Here the aim was to investigate the admixing effects of fine-root biomass, vertical distribution and morphology in *Pinus massoniana*–*Cinnamomum camphora* mixed plantations and corresponding monocultures at 10-, 24- and 45-year old stands.

**Results:**

The fine root biomass in the *Pinus*–*Cinnamomum* mixed forest exerted a certain degree of overyielding effect. These positive admixing effects, however, did not enhance with forest stand development. The overall relative yield total ranged from 1.83 and 1.51 to 1.33 in 10-, 24- and 45-year-old stand, respectively. The overyielding was mainly attributed to the over-performance of late successional species, *Cinnamomum*, in mixed stands. The vertical fine root biomass distribution model showed fine roots of pioneer species, *Pinus*, shifted to the superficial layer when mixed with *Cinnamomum.* Furthermore, the specific root length (SRL) of *Pinus* was significantly higher in *Pinus*–*Cinnamomum* mixed stands than that in monocultures, and the magnitude of differences increased over time. However, the vertical fine-root distribution and SRL for *Cinnamomum* did not show significant differences between monoculture and mixtures.

**Conclusions:**

Our results indicated that the magnitude of fine root overyielding in mixed forests showed a high degree of consistency with the total amount of fine root biomass itself, suggesting the overyielding effects in mixed forests were correlated with the degree of belowground interaction and competition degree involved. The late successional species, *Cinnamomum*, invested more carbon to belowground by increasing the fine root biomass in mixtures. While the pioneer species, *Pinus,* adapted to the presence of the species *Cinnamomum* by modification of vertical distribution and root morphological plasticity in the mixtures. These species-specific fine root foraging strategies might imply the differences of forest growth strategies of co-occurring species and contribute to the success and failure of particular species during the succession over time.

## Background

In the global carbon cycle, roots of forest trees are an important reservoir of carbon, which is an important component of C pool in terrestrial ecosystem and plays a vital role on global carbon flux and carbon library [1, 2]. In this context, in the past few decades, a lot of interests have been arose with fine root biomass and production in forest, since fine roots accounted for as much as one-third of global net primary productivity [3, 4] and are primary responsible for water and nutrient uptake by trees [5, 6]. Belowground interactions among co-occurring species play critical roles on the community structure and distribution of plants. The plants are even capable of recognizing non-self neighbours and tend to proliferate more roots into substrate shared with coexisting species, and likely resulting in rooting aggregation and overyielding, which is defined as higher production or biomass in mixture than that in the corresponding monocultures [7]. Therefore, mixed forests are considered to enhance ecosystem functioning and sustainability due to complementary resource use, environmental benefits and to be less susceptible to abiotic hazards like wind throw and snow break than pure stands [8, 9].

Most studies investigating tree species diversity effects on aboveground and belowground productivity in forests, however, were based on comparisons between two species mixtures with monocultures. For example, a number of studies found a higher fine root biomass and production in mixtures [10–13], although other studies showed otherwise [14, 15]. Recently, more studies were carried out to disentangle the effects of species diversity on the fine root biomass and production in mixed forests containing four to five tree species and the results were still equivocal [16, 17]. Notwithstanding, all these studies on the relationship between diversity and productivity were based on one particular growth stage, or one static stand age. To our knowledge, recently only one study has been conducted comparing the effects on tree species on fine root productivity at 8 and 34 years old [18]. Data on how fine root biomass, spatial distribution and morphology change at pure and mixed forests in relation to stand age, are comparatively few.

Fine root proliferation is greatly determined by environment conditions, such as nutrient supply in the soil, temperature and water. For example, Fine root vertical distribution is impacted by the spatial distribution of soil nutrients and moisture [19], as well as soil structure and bulk density [20]. Besides, with increasing forests development, the proportion of fine root biomass was prone to increase in the top soil [21] or indicate no change [2]. Furthermore, competition among individuals of the same species (intraspecific competition), as well as among different species populations (inter-specific competition), affects the process of tree root growth. Generally belowground competition depends on the soil exploitation capacity and exploitation efficiency of the fine-root systems of each plant, which were determined by the fine root biomass, surface area, root distribution within the soil horizons and specific root length (SRL) [22, 23]. Different plants within the community, in order to minimize competition for soil nutrients and moisture, may adjust the C investment to fine roots and distribution, and/or morphological traits to adapt to the competition. Previous studies showed that beech developed a more dynamic and adaptive fine root foraging strategies, e.g. biomass and vertical distribution, comparing to competitive species in mixed stands [24, 25]. However, how the belowground interactions may shift with forest development is not clear, which may mirror the aboveground competition.

Here the temporal changes of fine root biomass, vertical distribution and fine root morphology were investigated in *Pinus massoniana*–*Cinnamomum camphora* mixed plantations and corresponding single species plantations at age of 10, 24 and 45 years. Our objectives were to determine the magnitude of admixing effects on fine root biomass over time and to assess the possible shifts of foraging strategies for pioneer species (*P. massoniana*) and late successional species (*C. camphora*) along forest development. In this study we specifically tested the hypotheses that: (i) the total standing fine root biomass are higher in the mixed stands than those in corresponding monocultures, and the magnitude of positive admixing effect increases with forest development; (ii) the fine root foraging strategies of co-existing species, including fine root biomass, vertical distribution and morphological traits, in mixed forests mirrors the growth strategies of different species with forest development.

## Methods

### Field sites and experimental design

This study was carried out in two different sites in Hunan province, China. One area is located in the Botanical Garden in Changsha (28°06′N, 113°02′E). The annual rainfall on this site is 1422 mm and the mean annual temperature is 17.2 °C, belonging to typical subtropical monsoon climate. The altitude ranges from 50 to 100 m. The soil type is Alliti-Udic Ferrosols with well-drained clay-loam red soil developed from slate parent rock, and total N concentrations ranging from 0.57 to 1.56 g kg^−1^ in top 30 cm depth of the soil profile [26]. Single species and two species mixed patches, consisting of 24- and 45-year old *P. massoniana* were selected. In monocultures and mixed stand, few *Pinus elliotii* were also admixed here. Considering the similarity of growth characteristics and the difficulty of root separation between *P. massoniana* or *P. elliotii*, we treated them one group (thereafter called “*Pinus*”). Three plots of size 20 m × 20 m were established in mixed *Pinus*–*Cinnamomum* stands at age of 24 and 45 years old and corresponding pure species stands (*Pinus* and *Cinnamomum*) at each age, amounting to 18 plots. The another site is located in Taolin forestry station (28°55′N, 113°03′E) in Miluo county, approximate 100 km from the main site with similar climate and parent soil type. The mean annual precipitation is about 1353.6 and mean annual temperature is 16.9 °C. Here, only the smaller patches of mixed and pure species stands with 10 years old *Pinus* and *Cinnamomum* were found. Therein three plots of 12 m × 12 m in mixed forests stands and corresponding pure stands were set up as conducted above. Thereby our study consisted of 27 plots of mixed *Pinus*–*Cinnamomum* plantations and corresponding monocultures at age of 10, 24 and 45 years old. All the stems were recorded and selected site characteristics are presented in Table 1. More detailed information about the experimental site and soil condition referred to Wen et al. [26].

**Table 1:** Stand characteristics in pure species *Pinus* stands, pure *Cinnamomum* stands and mixed *Pinus*–*Cinnamomum* stands at the age 10, 24 and 45 years old (mean ± standard deviation)

Stand	Age	Species	Stand density (n ha^−1^)	Diameter at breast height (cm)	Height (m)	Basal area (m^2^ ha^−1^)
*Pinus* stands	10	*Pinus massoniana*	2592	9.38 ± 3.26	5.28 ± 3.97	20.06
	24	*Pinus massoniana*	2050	14.18 ± 4.34	12.86 ± 6.52	35.37
	45	*Pinus massoniana*	600	21.40 ± 5.30	12.47 ± 1.88	22.84
*Cinnamomum* stands	10	*Cinnamomum camphora*	2708	7.77 ± 2.60	5.99 ± 1.25	14.26
	24	*Cinnamomum camphora*	900	17.02 ± 6.52	13.71 ± 2.74	23.46
	45	*Cinnamomum camphora*	800	21.06 ± 6.73	13.24 ± 2.29	30.63
Mixed *Pinus*–*Cinnamomum* stands	10	*Pinus massoniana*	902	7.64 ± 1.82	4.73 ± 0.82	4.37
		*Cinnamomum camphora*	1689	8.14 ± 2.81	7.20 ± 0.73	9.83
	24	*Pinus massoniana*	267	19.88 ± 5.06	12.35 ± 1.64	7.80
		*Cinnamomum camphora*	592	15.27 ± 5.92	11.41 ± 3.13	12.45
	45	*Pinus massoniana*	250	19.69 ± 4.10	12.37 ± 2.60	7.91
		*Cinnamomum camphora*	325	20.94 ± 8.54	13.75 ± 2.79	12.91

### Fine root sampling and processing

The root sampling was carried out in April 2013. Six soil cores in each plot were taken randomly in each square plot by using soil steel auger (diameter of 10 cm) to the soil depth of 30 cm and sliced to three layers (0–10, 10–20, and 20–30 cm). A preliminary survey had shown that very few fine roots occurred below 30 cm soil depth here. All the samples were labeled and transferred to plastic bag, sealed, and transported to the laboratory in 4 °C refrigerator.

In the laboratory, the processes of root separation off the soil were conducted with floatation method [13, 17, 27]. All the roots were collected with sieve of 0.65 mm aperture. The washed fine roots were poured and suspended in water, then sorted to *Pinus* and *Cinnamomum*, live and dead ones visually according to morphological traits, turgescence, root elasticity, colour, periderm surface structure, and exposure degree of steles. As there were quite few other tree and understory species, the other species roots were discarded. Living roots of *Pinus* and *Cinnamomum* are intact, tough, and flexible. In contrast, dead roots were brittle and fractured easily and were distinguished by a dark to grey cortex and stele, or the complete loss of the stele and cortex. Live fine-root samples of each species were suspended in a water-filled transparent tray on a scanner (image resolution: 400 dpi) to facilitate root dispersing. The morphological characteristics of fine roots were analyzed using the root analysis system WinRHIZO 2013 (Regent Instruments Inc., Quebec, Canada) by using images obtained. Thereafter, the root samples were oven-dried at 60 °C to constant weight. The specific root length (SRL) (m g^−1^) was determined with the total root length by divided root dry weight.

### Data analysis

All data were tested for a normal distribution with the Shapiro–Wilk test. Analysis of variance (ANOVA) or a non-parametric Mann–Whitney *U* test was used to detect significant differences among three forest types. Differences between means were evaluated by Tukey’s test of honestly significant difference. To examine whether overyielding occurred, the relative yield total (RYT) was calculated as the ratio of expected fine root biomass in the mixture to the expected fine root biomass based on the adjusted fine root biomass per basal area in corresponding monocultures, as suggested by Lei [17] and Ma [28]. More specifically, the expected fine root biomass in mixture was calculated with formula.

$${\text{B}}_{\text{expected}} = \sum {({\text{B}}_{\text{i}} \times {\text{BA}}_{\text{i}} )}$$

where B_i_ is the observed fine root biomass per basal area of species i in pure stands, and BA_i_ is the basal area of species i in the mixture. The contributions of the different species, i.e. *Pinus* and *Cinnamomum*, to the relative yield total were calculated as the quotient of the fine-root biomass per basal area of each species at a particular stand age in the mixture to the counterpart value in the monoculture. RYT > 1 indicates overyielding on the stand level or for each component species. Significant differences from 1 were analyzed using *t* tests or the Mann–Whitney *U* test according to the data distribution.

To calculate the fine root vertical distribution, we adopted a commonly used equation developed by Gale and Grigal [29]:

$${\text{Y}} = 1-\upbeta^{\text{d}}$$

Therein, Y indicates the cumulative proportion of fine root biomass in the soil depth d (in cm). High values of β were indicate a large proportion of fine root at deeper soil depths, while low values indicate a large proportion of fine roots near the soil surface. Here we used β as criterion to compare fine root vertical distribution of *Pinus* and *Cinnamomum* in *Pinus*–*Cinnamomum* mixed forests and corresponding monocultures as different ages. All data analyses were conducted with R (R 3.0.3, R development Core Team, Vienna, Austria).

## Results

### Fine root biomass and overyielding

**Fig. 1:**
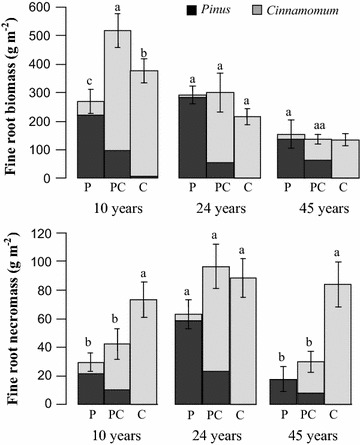
Live fine root biomass and fine root necromass in pure *Pinus* stand (P), *Cinnamomum* stand (C) and mixed *Pinus*–*Cinnamomum* stand (PC) in 0–30 cm soil depth at the age of 10, 24 and 45 years. Error bars indicate standard errors. Different letters indicate significant differences among different stands within the same ages (P < 0.05)

The ANOVA results indicated the stand age, soil depth and their interactions exerted significant influence on fine root biomass, while the effects of forest stand were not significant (Tables 2, 3). As the forests grows, the standing fine root biomass decreased with stand ages, averaged as 388.45, 269.27 and 138.59 g m^−2^ in 30 cm soil depth in 10-, 24- and 45-year-old stands, respectively. The standing fine root biomass was the highest in *Pinus*–*Cinnamomum* mixed stands compared to corresponding monocultures at 10- and 24-year stands in 30 cm depth soil profile (Fig. 1), but significant differences were only detected in 10-year-old forest stands. In mixed stands, *Cinnamomum* contributed more fine root biomass in the belowground part when compared with the aboveground abundance of mixed tree species. *Cinnamomum* accounted for the total fine root biomass 81.2, 81.3 and 53.2% in 10, 24 and 45-year-old *Pinus*–*Cinnamomum* mixed stands, respectively. Total fine root necromass showed lower values than fine root biomass and ranged from 17.84 to 96.54 g m^−2^ in the pure *Pinus*, *Cinnamomum* and mixed *Pinus*–*Cinnamomum* stands at differ ages (Fig. 1). The standing fine root necromass was the highest in the pure *Cinnamomum* stands at 10- and 45-year stands compared with mixed stands at the corresponding age in 30 cm depth soil profile, which differed significantly from each stands (P < 0.05) (Table 2). In 24-year-old stands, however, the mixed stands showed the highest fine root necromass.

**Table 2:** The effects of plantation stand and stand age on fine root biomass, fine root necromass and specific root length using a two-way analysis of variance

Factor	Fine root biomass			Fine root necromass			Specific root length		
	df	F value	P	df	F value	P	df	F value	P
Stand	2	1.78	0.172	2	7.99	< *0.0001*	1	9.77	*0.0021*
Age	2	23.06	< *0.0001*	2	9.90	< *0.0001*	2	1.72	0.1824
Stand × age	4	2.97	*0.0217*	4	1.80	0.1318	2	2.53	0.0823

Italic font indicates significant differences at *P* < 0.05

**Table 3:** The effects of plantation stand, stand age and soil depth on fine root biomass using a three-way analysis of variance

Factor	df	SS	F value	P
Stand	2	2.37	2.00	0.1361
Age	2	30.68	25.91	< *0.0001*
Depth	2	43.56	36.79	< *0.0001*
Stand × age	4	7.91	3.34	*0.0105*
Stand × depth	4	1.91	0.80	0.5228
Age × depth	4	11.19	4.72	*0.0010*
Stand × age × depth	8	1.88	0.40	0.9225

Italic font indicates significant differences at *P* < 0.05

**Fig. 2:**
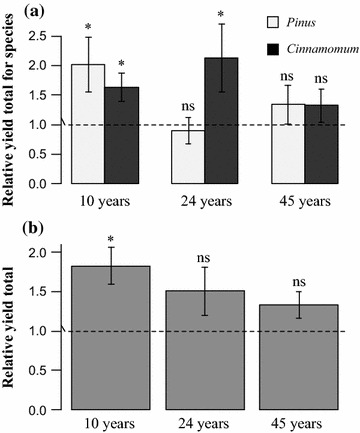
Relative yield total for each species (**a**) and relative yield total for total standing fine root biomass (**b**) and in *Pinus*–*Cinnamomum* mixed stands at ages of 10, 24, and 45 years old stand age in comparison to monocultures (reference level = 1). Asterisks denote significant differences from 1 with t test or Mann–Whitney *U* test, *P* < 0.05. Each datum shows the mean ± SE

Relative yield total for each species in mixed plantations were calculated based on fine root biomass per basal area. The results showed RYT of *Cinnamomum* was bigger than 1 in mixed plantations at all stand ages. Among them, 10- and 24-year-old forest stands showed a relative yield total value for fine root biomass that were significantly different from one (P < 0.05). For *Pinus,* the RYT showed inconsistent pattern that RYT was higher than one in 10- and 45-year-old mixed stands, while the RYT was marginally lower than one in 24-year-old stand (Fig. 2a). On the stand level, the overall RYT were bigger than one in all the three development stages, but the values of RYT seemed to decline with increasing stand ages, averaged as 1.83, 1.51 and 1.33 in 10-, 24- and 45-year-old stand, respectively (Fig. 2b).

### Vertical fine root distribution

**Fig. 3:**
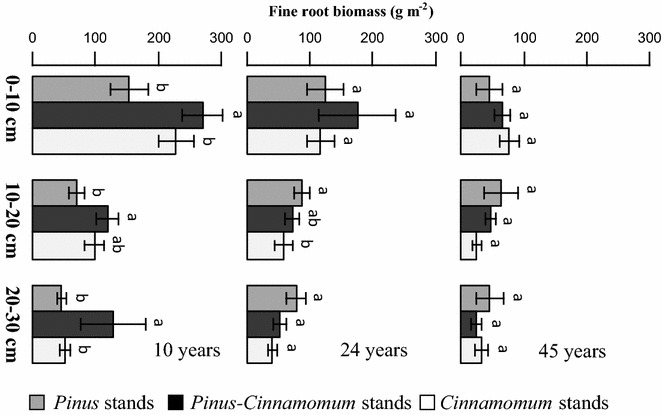
Fine root biomass in pure *Pinus*, *Cinnamomum* and mixed *Pinus*–*Cinnamomum* stands in 0–10, 10–20 and 20–30 cm soil depth at the age of 10, 24 and 45 years. Error bars indicate standard error. Different letters indicate significant differences among different stands within the same soil profile and age stages (*P* < 0.05)

**Fig. 4:**
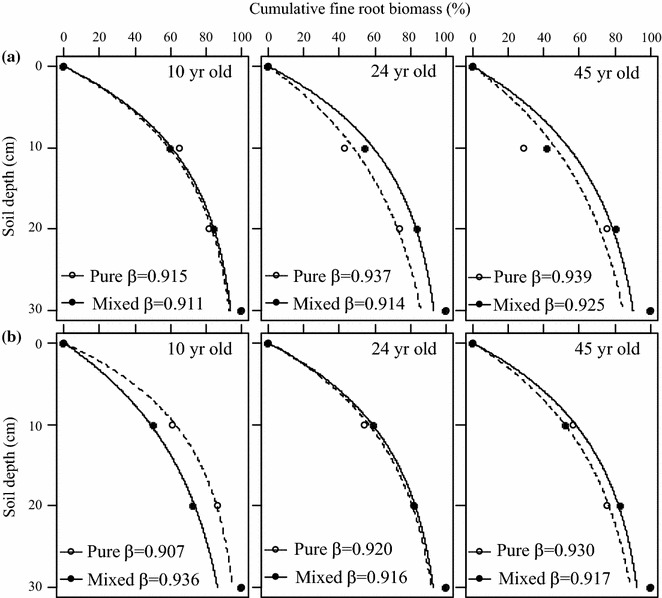
Cumulative fine root biomass along the soil profiles and the coefficients of the rooting distribution (β) for *Pinus* (**a**) and *Cinnamomum* (**b**) in the pure and mixed stands at ages of 10, 24 and 45 years. The β value indicates the degree of fine root biomass decreases with soil depth

The standing fine root biomass decreased gradually with soil depth, and fine root biomass was highest in 0–10 cm for almost all the cases in the whole stand ages, which accounted for 56.0, 51.7 and 47.2% in 10-, 24- and 45-year-old stands. When compared with parallel forest stand within the same soil profiles, the fine root biomass was highest in the *Pinus*–*Cinnamomum* mixed stands in all the three soil layers in 10-year-old stands and in the top soil layer in 24-year-old stands. But the significant differences were detected only in 10-year-old stands (P < 0.05) (Fig. 3). The abundance of species fine roots declined exponentially with increasing soil depth in the pure and mixed stands. Furthermore, the simulated rooting vertical distribution model of β value for *Pinus* and *Cinnamomum* growing in pure stands at different stand ages showed the similar patterns that both species allocated more fine roots to the deeper layer with increasing stand ages. Compared the β values for *Pinus* and *Cinnamomum* in the pure and mixed stands, however, the results showed that the adjusted β value for *Pinus* in pure stands were significantly higher than that in the mixed stands at all forest ages (see Fig. 4). The regression fitting results showed that the β value was 0.915, 0.937, 0.939 in pure stands, and 0.911, 0.914, 0.925 in mixed stands with quite promising coefficients of determination (r^2^ > 0.95, P < 0.01) in forests age of 10-, 24-, and 45-year old, respectively, suggesting fine roots o*f Pinus* were more concentrated in the top soil when mixed with *Cinnamomum*. For *Cinnamomum*, the β values did not show consistent pattern with increasing stand age as it increased in the pure stands, but decreased in the mixed stands along chronosequence.

### Specific root length

**Fig. 5:**
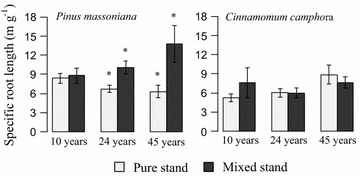
Comparisons of specific root length for live fine root of *Pinus massoniana* and *Cinnamomum campho*ra in pure and mixed stands at the age of 10, 24 and 45 years. Error bars indicate standard errors. Asterisks indicate significant differences between pure and mixed stands for the specific species within the same age stages (P < 0.05)

The comparison of specific root length (SRL) in the pure and mixed stands revealed striking differences for *Pinus* and *Cinnamomum*. The SRL of *Pinus* in mixed stands were significantly higher than those of corresponding pure stand, and the differences became more pronounced over time. For *Cinnamomum*, however, structural trait did not show regular pattern in pure and mixed stand plots. Result from one-way ANOVA revealed that both plantation type and stands age had significant effects on morphology. At in the pure *Pinus* stand, the SRL changed along the chronosequence, decreasing from 8.84 m g^−1^ in the 10-year old stands to 6.72 m g^−1^ in the 24-year old stands and 6.29 m g^−1^ in the 45-year old stands. However, the SRL of mixed stands increased along chronosequence. And significant differences were only detected between the 24- and 45-year old stands (P < 0.05) (Fig. 5a). For species *Cinnamomum*, the SRL in the pure stands and mixed stands was basically similar, ranging from 5.24 to 8.90 m g^−1^ (Fig. 5). Comparing with different age stages, the results showed that SRL of *Cinnamomum* was increased with increase stand age in the pure stands. However, this kind of phenomenon did not show in the *Pinus*–*Cinnamomum* mixed stands.

## Discussion

In this study, the fine root biomass seemed to decrease both in pure and mixed stands with increasing stand age, which is likely related to the decreased stand density with development stages here. The high tree density may have accounted for the higher root biomass, which is in agreement with a previous report described for 13-year-old postfire lodgepole pine forests, where the fine root biomass increased with tree density [30]. In contrast to previously published studies that maximum fine root biomass is reached at the canopy closure stage of stand development [2, 31, 32], we found early stand developmental stage with high root biomass. The previous study showed that fine root biomass reached a maximum at an approximate age of 25 years, and then declined to a steady-state, as forests approached maturity. In our study sites, the canopy was already closed, even in the 10-year-old stand due to high planting density. Besides stand ages, the fine root biomass dynamics might also relate to the other stand characteristics, such as tree density, stand structure, basal area and aboveground biomass, etc. [33–35].

When compared with three types of plantations, the fine root biomass was higher in the *Pinus*–*Cinnamomum* mixed stands than those in the pure *Pinus* and *Cinnamomum* stands in 30 cm soil depth in 10- and 24-year-old stands. Many previous studies reported the similar pattern that species-rich forests exhibited higher fine root biomass than species-poor stands [12, 36]. Although the absolute fine root biomass in the *Pinus*–*Cinnamomum* mixed forests were not significantly higher than that the counterpart monocultures in 24-year-old, and even lower than that in monocultures in 45-year-old stands, the RYT was higher than one, suggesting overyielding when comparing the adjusted fine root biomass per basal area in mixed forest with monocultures (Fig. 2b) [37]. Likewise, fine root overyielding was reported in mixed forests of *Eucalyptus grandis* and *Acacia mangium* stands [38], as well as in European beech, sessile oak, Norway spruce and Douglas fir mixed stands at two-, three-, and four-species neighbourhoods in comparison to monocultures [17].

Here we primarily attempted to assess the variations of these admixing effects over time and expected that the admixing effect would be more pronounced over time, as the interactions between different species are assumed to be more intense over time. On the contrary, in this study, the magnitude of over-yielding in the *Pinus*–*Cinnamomum* mixed forests declined with stand development as shown in Figs. 1 and 2. The significant differences between the *Pinus*–*Cinnamomum* mixed forests and corresponding monocultures were only detected in 10-year-old forests. Besides, the direct evidence showed that relative yield total decreased from 1.83, and 1.51 to 1.33 in 10-, 24- and 45-year-old stand, respectively. This pattern is consistent the trend that the standing fine root biomass decreased with stand. Therefore, it is likely that the magnitude of fine root overyielding in mixed forests was correlated with fine root biomass and the belowground competitive degree involved instead.

Fine-root turnover contributes substantially to soil organic matter inputs and productivity in forest ecosystems [39]. Thus, understanding the turnover of fine-roots is important to unveil the belowground ecosystem function. The ratio of live to dead roots (i.e., B/N) could reflect the turnover characteristics of fine roots [36]. In our study, the B/N ratio of *Pinus* was higher than that of *Cinnamomum*. This could be attributed to a faster decomposition rate or a lower mortality of coniferous species *Pinus* compared to broadleaved species *Cinnamomum* [40].

Here we calculated the RYT for the component species in the mixtures to estimate the specific performance and dynamics of each species to the overyielding in the mixtures over time, and foraging strategies as well. In the mixtures, the RYT of late successional species, *Cinnamomum,* was higher than one for all the stand development stages and the differences were significant from one in 10- and 24-year-old stands. This pattern was supported by data on fine root biomass, which *Cinnamomum* accounted for 81.2, 81.3 and 53.2% in 10, 24 and 45-year-old *Pinus*–*Cinnamomum* mixed stands, respectively. Figure 1 suggesting *Cinnamomum* invested more carbon to belowground fine roots when co-occurring species presents. In contrast, the pioneer species, *Pinus,* showed significant higher RYT only in 10-year-old stand and then fluctuated from one thereafter.

In our study, we compared the vertical distribution of fine root biomass with the exponential model of Gale and Grigal [29] and found very similar patterns for the overall distribution in pure stands along chronosequence. Two species showed the exponential indicator (β values) increased with stand development. The β value indicated that there was clear spatial separation of the fine root systems of the *Pinus* within 30 cm soil profile. The *Pinus* roots occupied the deeper soil layers in the pure stand whereas it shifted to the superficial layers when mixed with *Cinnamomum*. Many previous researches revealed significant effect of mixed stand on fine root distributions. Bolte and Villanueva [14] suggested that fine root of *Picea abies* distributed deeper in mixed stands than pure stands. Moreover, in mixed stand of *Fagus sylvatica* and *Quercus Petraea*, fine roots of *F. sylvatica* grew deeper than fine roots of *Q. petraea* [41]. The presence of *Cinnamomum* in the mixed stands could have pushed the fine root system of *Pinus* towards the soil surface where the water and nutrient were more enriched. In mixed stand fine roots tend to proliferate and compete with neighbors for nutrients and water by developing a more flexible fine root system when there is more intense belowground competition [42, 43]. On the other hand, the shallower root allocation could be more susceptible to the drought. For *Cinnamomum*, the fine root distribution model indicated that there was no obvious spatial separation of the fine root systems within 30 cm soil profile. Therefore, different tree species may have different strategies for the presence of neighbor species in terms of vertical niche separation.

The specific root length (SRL) was used as indicator for nutrient uptake efficiency and responses to environmental changes or competition [44]. SRL can reflect the root growth strategies on the efficiency of consumption photosynthetic primary product, high SRL indicate high efficiency of using photosynthetic primary product of plant root systems [45]. Our studies showed that SRL of *Pinus* decreased slightly with increase forest age in monocultures, but increased with stand age in mixtures. In 45-year-old stand, the SRL of *Pinus* in the *Pinus*–*Cinnamomum* mixed stand was up to two-fold higher than that in monocultures, suggesting *Pinus* exploited water and nutrient resources more efficiently with given carbon investment when growing admixed with *Cinnamomum*. It is likely that pioneer species, *Pinus*, was stressed by the competition from the later successional species, *Cinnamomum.* To what extent the pioneer species can adjust the root morphology to the neighbour species competition could be a big issue. The results were consistent with previous study showing that the specific root length (SRL) and specific root area (SRA) for beech were higher in beech–spruce mixtures than that in monocultures [14]. For *Cinnamomum,* in contrast, fine root morphology was rather similar in pure and mixed stands. The SRL of *Cinnamomum* seemed to increase along chronosequence, but no significant differences were detected, in agreement of previous study that mean SRL was not significantly different among the *F. sylvatica*, *Quercus robur* and *Alnus glutinosa* chronosequences [46].

Furthermore, fine-root morphological traits may vary as a consequence of interactions with soil biota, such as ectomycorrhizal fungi, which may confound root plasticity responses to resource availability [47]. This is especially true for pine species which are obligatedly ectomycorrhizal. Unfortunately, we have no ectomycorrhizal data to examine whether this ectomycorrhizal infection effect is mirrored morphological plasticity or otherwise, which merits further investigations.

## Conclusions

Here our experiment detected the variations of admixing effect of fine root biomass with forest development. The results showed fine root overyielding in mixed plantations and this positive admixing effects did not increase over time, instead the magnitude of overyielding in mixed forests was correlated with the degree of belowground interaction and competition degree involved. In 10-year-old stands, two species showed significant admixing positive effects on fine root biomass, while in 24- and 45-year-old stands, the overyielding was mainly attributed to the overperformance of late successional species *Cinnamomum*, which invested more carbon to belowground by increasing the fine root biomass in mixtures, suggesting that the duration time of over-yielding of *Cinnamomum* roots was probably longer than *Pinus*. While the pioneer species, *Pinus,* adapted to the presence of the species *Cinnamomum* by modification of vertical distribution and root morphological plasticity in the mixtures. The contrast performances of root foraging strategies between early and late successional species by using either extensive adaptation strategy for late successional species *Cinnamomum* to increase carbon investment into fine root biomass, or intensive adaption strategy for pioneer species *Pinus* to shift fine root distribution to the top soil layer and to increase SRL in mixtures. These species-specific fine root foraging strategies might imply the differences of forest growth strategies of co-occurring species and likely contribute to the success or failure of particular species during the succession over time.
